# Impact of Repeated Stress on Traumatic Brain Injury-Induced Mitochondrial Electron Transport Chain Expression and Behavioral Responses in Rats

**DOI:** 10.3389/fneur.2013.00196

**Published:** 2013-12-12

**Authors:** Guoqiang Xing, Erin S. Barry, Brandi Benford, Neil E. Grunberg, He Li, William D. Watson, Pushpa Sharma

**Affiliations:** ^1^Department of Anesthesiology, Uniformed Services University of the Health Sciences, Bethesda, MD, USA; ^2^Department of Medical and Clinical Psychology, Uniformed Services University of the Health Sciences, Bethesda, MD, USA; ^3^Department of Psychiatry, Uniformed Services University of the Health Sciences, Bethesda, MD, USA; ^4^Department of Neurology, Uniformed Services University of the Health Sciences, Bethesda, MD, USA

**Keywords:** oxidative phosphorylation, mitochondria, electron transport chain, behavior change, TBI, PTSD

## Abstract

A significant proportion of the military personnel returning from Iraq and Afghanistan conflicts have suffered from both mild traumatic brain injury (mTBI) and post-traumatic stress disorder. The mechanisms are unknown. We used a rat model of repeated stress and mTBI to examine brain activity and behavioral function. Adult male Sprague-Dawley rats were divided into four groups: Naïve; 3 days repeated tail-shock stress; lateral fluid percussion mTBI; and repeated stress followed by mTBI (S-mTBI). Open field activity, sensorimotor responses, and acoustic startle responses (ASRs) were measured at various time points after mTBI. The protein expression of mitochondrial electron transport chain (ETC) complex subunits (CI-V) and pyruvate dehydrogenase (PDHE1α1) were determined in four brain regions at day 7-post mTBI. Compared to Naïves, repeated stress decreased horizontal activity; repeated stress and mTBI both decreased vertical activity; and the mTBI and S-mTBI groups were impaired in sensorimotor and ASRs. Repeated stress significantly increased CI, CII, and CIII protein levels in the prefrontal cortex (PFC), but decreased PDHE1α1 protein in the PFC and cerebellum, and decreased CIV protein in the hippocampus. The mTBI treatment decreased CV protein levels in the ipsilateral hippocampus. The S-mTBI treatment resulted in increased CII, CIII, CIV, and CV protein levels in the PFC, increased CI level in the cerebellum, and increased CIII and CV levels in the cerebral cortex, but decreased CI, CII, CIV, and PDHE1α1 protein levels in the hippocampus. Thus, repeated stress or mTBI alone differentially altered ETC expression in heterogeneous brain regions. Repeated stress followed by mTBI had synergistic effects on brain ETC expression, and resulted in more severe behavioral deficits. These results suggest that repeated stress could have contributed to the high incidence of long-term neurologic and neuropsychiatric morbidity in military personnel with or without mTBI.

## Introduction

Estimates as high as 24% of U.S. military personnel returning from Iraq and Afghanistan battlefields have suffered from a mild traumatic brain injury (mTBI) and/or post-traumatic stress disorder (PTSD) (1–5). Comorbidity of mTBI and PTSD are also high among this sub-population (3). Despite the numbers of cases, dedicated resources and research, and marked concern about these conditions, minimal progress has been made toward understanding the biological mechanisms producing these pathologies.

The brain is the organ of glucose metabolism and adenosine triphosphate (ATP) utilization, which expresses our essence, our nature, and is solely responsible for our behavioral and psychological functions to help define who we are. Dysregulated brain energy metabolism (i.e., acute hyperglycemia during the early phase of TBI and subsequent hypoglycemia during the chronic phase of TBI) is a metabolic characteristic of TBI that is associated with symptom severity and poor prognosis for functional recovery from TBI (6–9).

Pyruvate dehydrogenase (PDH) is the rate-limiting enzyme that irreversibly transfers the glycolysis product pyruvate into acetyl-coenzyme A (CoA) for efficient production of nicotinamide adenine dinucleotide (NADH) and ATP through mitochondrial tricarboxylic acid (TCA) cycle and oxidative phosphorylation pathway (OXPHOS). OXPHOS is mediated by mitochondrial electron transport chain (ETC) complex subunits (CI, CII, CIII, CIV, and CV).

Mitochondrial ETC generate ATP by coupling electron transfer between electron donors (i.e., NADH) and the electron acceptor (O_2_) with the transfer of protons (H^+^) across the membrane to generate energy in the form of ATP. Complex I (CI, NADH dehydrogenase) and Complex II (CII, succinate dehydrogenase flavoprotein) accept and transfer electrons to coenzyme Q which sequentially transfers electrons to Complex III (CIII, cytochrome reductase), cytochrome *c*, and Complex IV (CIV, cytochrome oxidase), where oxygen is the terminal acceptor. Complex V (CV) couples proton gradients to ATP synthesis, allowing proton flow from inter-membrane space to the matrix via a special enzyme, converting ADP to ATP.

We previously reported that the PDH pathway is altered in animal models of TBI (10–13). Complex abnormalities in ETC have also been found in many neurological diseases (14–16), yet its potential involvement in combined TBI and stress is unknown. Because aberrant ETC complex activities are the primary source of intracellular reactive oxygen species (ROS), alterations in ETC complexes could lead to increased ROS production, inflammation, impaired signal transduction, mitochondrial damage, and cell death, thus compromising brain vulnerability to subsequent stress and injuries.

Brain energy metabolism is also disrupted during traumatic stress. Under life-threatening situations, energy reserves are intensively mobilized for fight-or-flight response via sympathetic activation and epinephrine/norepinephrine release. This reaction increases cardiovascular output, aerobic supply, fear memory, visual and auditory sensitivity, alertness, vigilance, and selective attention that are essential for survival (17–19). However, potential metabolic over-reactivity meant to increase survivability in an emergent situation has long-lasting deleterious effects that may compromise the brain’s response to subsequent injuries.

The main goal of this study was to determine if prior repeated stress altered TBI-induced brain ETC expression and behavioral functions. We hypothesized that the combination of repeated stress followed by mTBI could affect brain ETC complex expression in a way that is different from repeated stress or mTBI alone. We included measures of behavioral and psychological unconditioned responses (i.e., activity, sensorimotor responses, acoustic startle reflexes, and measures of depression-related behavior) to determine whether the repeated stress has functional effects in addition to changes to mitochondrial expression. Rat fluid percussion (FP) was used to create mTBI and repeated stress was used to model PTSD.

## Materials and Methods

### Animals and experimental groups

Adult, male Sprague-Dawley rats (175–275 g) were obtained from Harlan Laboratories (Indianapolis, IN, USA). All procedures were performed in accordance with guidelines of the National Institutes of Health and were approved by the Institutional Animal Care and Use Committee (IACUC) at the Uniformed Services University of the Health Sciences (USUHS). Rats were pair housed in standard polycarbonate shoebox cages (42.5 cm × 20.5 cm × 20 cm) with hardwood chip bedding (Pine-Dri) and kept on a 12-h reversed light-dark cycle, with food (Harlan Teklad 4% Mouse/Rat Diet 7001) and water continuously available. All animals were weighed just before the experiment (T0) and at the end of the experiment (T7) as a measure of their general health. Rats were given coded tail numbers and assigned randomly to experimental groups after 1 day of acclimation to the environment [Naïve, Stress, mTBI, and mTBI with prior stress (S-mTBI)]. For behavioral analyses, a total of 54 rats were used in this experiment (Naïve = 16; Stress = 12; mTBI = 16; S-mTBI = 10). In the case of the S-mTBI animals, the stress was given for 3 consecutive days and then mTBI was administered within 24 h. This group originally had 12 animals, but 2 died during the TBI surgery. Necropsies of these animals by Laboratory of Animal Medicine (LAM) personnel found no identifiable cause, including infection, for these deaths. At day 7-post injury, animals were sedated under isoflurane anesthesia, and tissues were collected. Brain tissue from eight animals of each treatment group was dissected for biological analysis.

### Induction of fluid percussion injury

Mild traumatic brain injury was induced in rats according to our published procedure (13). In brief, animals were anesthetized with 1–3% isoflurane in oxygen. Under sterile conditions, a 3-cm sagittal incision was made along the midline to expose the cranium. A 5-mm burr hole was placed 2 mm to the right of the sagittal suture halfway between bregma and lambda using a 5-mm trephine drill bit exposing the dura. A Luer-Lock needle hub was placed into the burr hole and cemented to the cranium using cyanoacrylate. The glue was allowed to completely dry, and the empty Luer-Lock hub was filled with normal saline before being connected to the TBI device. A FP pulse of 2.5 atm was administered by an injury cannula positioned parasagittally over the right cerebral cortex. The FP pulse was administered by a pendulum modulated FP biomechanical device (Richmond, VA, USA). The Luer-Lock hub was removed and defects in the cranium were repaired with bone wax. The skin was closed with a surgical skin stapler. Animals were allowed to stabilize in the warm blanket before returned to their home cages. At 7 days post mTBI, animals were sacrificed under anesthetization, and brains were removed followed by the dissection of prefrontal cortex (PFC), cerebellum, and the ipsilateral and contralateral of mTBI hippocampus and cerebral cortex.

### Repeated tail-shock stress procedure

This paradigm was chosen because it has previously demonstrated that repeated immobilization and tail-shock stress sessions are more effective than a single stress session in producing physiological and behavioral abnormalities, such as elevations in basal plasma corticosterone levels and delayed exaggerated acoustic startle responses (ASRs) are similar to symptoms observed in PTSD patients (20–25). This restraint tail-shock model of stress in rats is adapted from the “learned helplessness” paradigm in which animals undergo an aversive experience under conditions in which they cannot perform any adaptive response (26). The stress procedure consisted of a 2-h per day session of immobilization and tail shocks over 3 consecutive days. Stressed animals were restrained in a Plexiglas tube and given 40 electric tail shocks (2 mA, 3 s duration) at varying intervals (140–180 s). Animals were returned to their home cages immediately after exposure to the stress procedure.

### Animal behavioral measures

Behavior was observed during the animals’ dark cycle (i.e., during their active period). All animals underwent behavioral evaluation prior to stress and/or injury (baseline), and at two other time points during the week after injury. Behavioral measures included: open field activity (OFA) to measure general health and depression-related behaviors; neurobehavioral testing [revised neurobehavioral severity scale (NSS-R)] to measure sensory-motor functioning; and ASR with and without pre-pulse to measure startle and attention.

### Open field activity

Open field activity was measured using Accuscan Superflex Sensor Version 2.2 infrared photocell system in the Accuscan Instruments testing chamber (measuring 40 cm × 40 cm × 30 cm; Accuscan Instruments Incorporated, Columbus, OH, USA) located in a dedicated room designed to minimize acoustic interruptions. The testing chamber was constructed of Plexiglas with a ventilated, removable Plexiglas lid that prevents the animal’s escape during the trial but allows adequate airflow. The animal’s locomotion was captured by three, paired 16-photocell Superflex Sensors, which transmit the location data to the Accuscan Superflex Node which was processed and aggregated by Accuscan Fusion Software (Version 3.4). Animals were acclimated to the chambers prior to the beginning of the experiment. They then received a baseline measurement prior to injury and/or stress and were measured at days 3 and 5 post injury. The OFA of each rat was measured for 1 h during its active period (dark cycle).

### Revised neurobehavioral severity scale

The NSS-R is a specific, continuous sequence of behavioral tests and observations that is a sensitive and reliable measure in rodents (27, 28). This measure was originally designed to model a clinical neurological exam conducted in patients. This particular sensory-motor assessment scale was based on several previous reports (29–32) and has been modified to increase standardization. The tests assess reflex suppression, general movement, and postural adjustments in response to a challenge. The NSS-R uses a three-point Likert scale, in which a normal, healthy response is assigned a “0,” a partial or compromised response is assigned a “1,” and the absence of a response is assigned a “2.” This three-point scale is clear and reliable and allows for greater discrimination based on sensory-motor responses than do previous scales that used two-point ratings of each response. The NSS-R has a scoring range of 0–20 with higher scores reflecting greater extent of injury. Three NSS-R sessions were conducted in this experiment as a within-subject measure: one before stress/injury (baseline) and two after injury (days 3 and 5).

### Acoustic startle response with and without pre-pulse

Acoustic startle responses with and without pre-pulse were measured in a Med Associates Acoustic Response Test System (Med Associates, Georgia, VT, USA). The Test System consists of weight-sensitive platforms inside individual sound-attenuated chambers. Responses were recorded by an interfaced Nexlink computer. Each rat was placed individually in a ventilated holding cage (small enough to restrict extensive locomotion but large enough to allow the subject to turn around and make other small movements) on the weight-sensitive platform. Following placement of animals in the chambers, a 3-min acclimation period was conducted, in which no startle stimuli were presented. Startle stimuli consisted of 110 or 120 dB noise bursts of 20 ms duration sometimes preceded by a 100-ms, 68 or 82 dB, 1 kHz pure tones (pre-pulses). Intensity of sound in decibel was verified by a Larson-Davis Sound Pressure Machine Model 2800 (unweighted scale; re: 0.0002 dyn/cm^2^). Each startle stimulus had a 0-ms rise and decay time so that onset and offset were abrupt. There were multiple types of stimulus trials, and each trial type was presented six times and averaged. Trial types were presented in random order to avoid order effects and habituation. Animals’ movements in response to stimuli were measured as a voltage change by a strain gage inside each platform. Animals were acclimated to the chambers twice prior to the beginning of the experiment. They then received a baseline measurement prior to injury/stress and were measured at days 4 and 6 post injury.

### Western blot

Brain tissue homogenates from four brain regions (prefrontal cortex, cerebellum, hippocampus, and cerebral cortex) were homogenized and sonicated in the T-Per tissue lysis buffer (Pierce, IL) in the presence of protease inhibitor cocktail (Sigma, St. Louis). For the mTBI and S-mTBI animals, ipsilateral and contralateral hippocampus, and cerebral cortex were dissected and processed respectively. Protein concentrations were determined using a Bradford assay (BioRad, CA, USA). Aliquots of 20 μg proteins were separated by electrophoresis on NuPage Novex Midi Bis-Tris gels (4–12%) and transferred to a polyvinylidene difluoride membrane (PVDF), Millipore. The membranes were rinsed in a 0.01-M Tris-buffered saline (TBS) solution (pH 7.4, 0.1% Triton X-100) for 30 min, blocked in 5% bovine serum albumin for 30 min, and incubated overnight at 4°C with the primary mouse monoclonal antibodies for ETC complex subunits CI-V and PDHE1α1 (Abcam, UK) at a dilution of 1:200, each in a TBS solution containing 3% bovine serum albumin. The membranes were then washed three times with TBS solution for 30 min and incubated at room temperature with a horseradish peroxidase-conjugated secondary anti-mouse antibody (1:5000 dilution) in TBS solution for 60 min.

Due to the lack of an appropriate housekeeping mitochondrial protein (33, 34), WB band intensity was expressed as fold change relative to naives, but was not normalized to an internal control. However, we took extra steps to normalize our data by (1) loading the equal amount of protein for each sample; (2) all samples were processed, loaded, and run in parallel, and (3) transfer efficiency of proteins to the PVDF membrane was confirmed by staining with Ponceau solution. Immunoreactive bands were visualized using enhanced chemiluminescence Western blotting detection reagents (GE Healthcare Bio-Sciences Corp, Piscataway, NJ, USA). The western blots were captured with a digital camera and the intensities of protein bands were quantified with NIH Image 1.62.

### Statistical analysis

For behavioral data, repeated measures analyses of variance (rANOVA; to assess for overall main effects for Time, Group, Injury, Drug, and any interactions) and analysis of variance (ANOVA; to assess for main effects of Group, Injury, Drug, and any interactions at each time point) were conducted for each of the dependent variables. Baseline measurements were used as a covariate to account for any baseline differences. Pairwise comparisons were performed where appropriate. OFA scores were separated into two subscales: horizontal activity (HA) and vertical activity (VA). Analyses for all measures except for OFA included data for all subjects (*N* = 54). The OFA included a subset of the subjects (*N* = 46) because of an equipment malfunction during one cohort of eight subjects. Cohorts were similar among experimental groups; therefore, the remaining data are representative of all experimental conditions. All tests were two tailed using α = 0.05. Data analyses were performed at the conclusion of the project, after all measurements were collected.

Mitochondrial complex I-V and PDHE1α1 protein expression levels were analyzed for each brain region (prefrontal cortex, cerebellum, and contralateral and ipsilateral hippocampus and cerebral cortex) using a one-way ANOVA followed by LSD multiple comparison. A *p*-value <0.05 was considered statistically significant.

## Results

In reference to the weight gain in the groups the following was observed. The mTBI animals gained only 2.7% of the baseline weight (*p* < 0.05), while the stressed animals gained significantly more both with mTBI at 10.8% and without mTBI at 11.9% (*p* < 0.001). Naïve animals also gained a significant amount of weight at 7.5% in comparison to the baseline, but less than the stressed rats. The behavioral and western blot analysis quantification was not conducted in a blinded fashion.

### Behavioral functional outcomes

#### Open field activity

**Figure 1:**
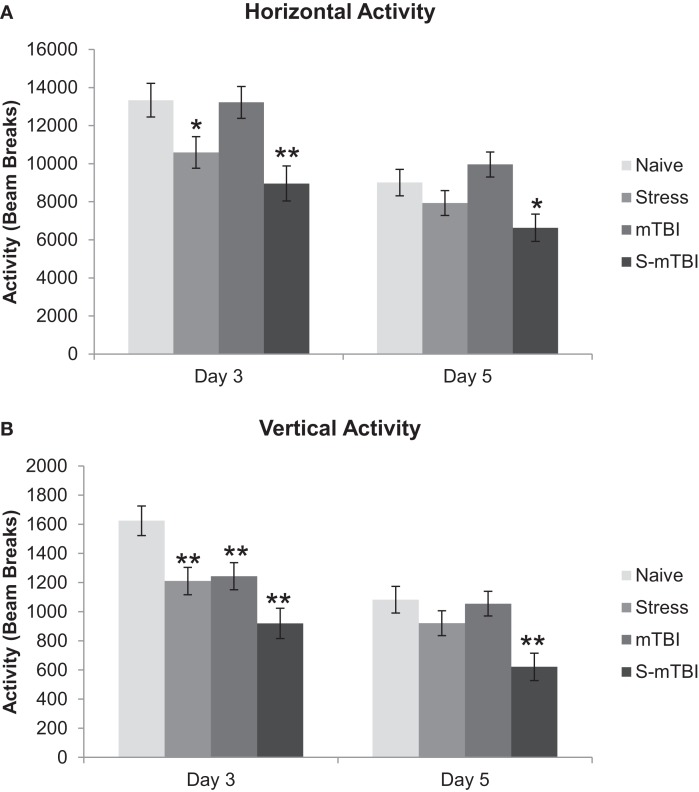
**Effects of stress, mTBI, or the combination on open field activity**. Activity was measured for 60 min at baseline (BL), and 3 and 5 days post injury and the number of beam breaks was collected. **(A)** Horizontal activity (measure of general health and movement) of the animals throughout the experiment. **(B)** Vertical activity (measure of depression-related behavior) of the animals throughout the experiment. Vertical activity measurement post injury was covaried for baseline measurements due to differences at baseline between groups. *p < 0.05, **p < 0.01 vs. Naïves, respectively.

Open field activity measures naturally occurring behaviors exhibited when an animal explores and interacts with its surroundings. These measures provide reliable and valuable data about gross motor and specific movements related to psychological conditions such as anxiety-related and depressive-related behaviors (35–38). For the purposes of this experiment, two variables were extracted from the animal’s movement within the chambers: HA and VA. Figure 1A presents HA (an index of general health and movement) of the animals. Overall, there was a main effect for Group, *F*(3,40) = 6.00, *p* < 0.01, η^2^ = 0.31, such that Naïve animals had significantly more activity overall than did the S-mTBI animals. There was a main effect for Stress, *F*(1,40) = 16.25, *p* < 0.001, η^2^ = 0.29, such that the non-stressed animals had significantly more activity overall than did the stressed animals. There also was a significant Time × Stress Interaction, *F*(1,40) = 4.13, *p* < 0.05, η^2^ = 0.09. At 3 days post injury, there was a main effect for Group, *F*(3,40) = 5.83, *p* < 0.01, η^2^ = 0.30, such that the Naïve animals had significantly more activity than did the Stress animals and the S-mTBI animals. There also was a main effect for Stress, *F*(1,40) = 16.38, *p* < 0.001, η^2^ = 0.29, such that the non-stressed animals had significantly more activity than did the stressed animals. At day 5 post injury, there was a main effect for Group, *F*(3,40) = 4.38, *p* < 0.01, η^2^ = 0.25, such that Naïve animals had significantly more activity than did S-mTBI animals. There also was a main effect for Stress, *F*(1,40) = 10.58, *p* < 0.01, η^2^ = 0.21, such that the non-stressed animals had significantly more activity than did the stressed animals.

Figure 1B presents VA (an index of depression-related behaviors; where less VA indicates more depression-related behaviors) of the animals. Overall, there was a main effect for Group, *F*(3,40) = 6.46, *p* < 0.001, η^2^ = 0.32, such that Naïve animals had significantly more VA than did S-mTBI animals and Stress animals. There was a main effect for Injury, *F*(1,40) = 7.91, *p* < 0.01, η^2^ = 0.16, such that non-injured animals had significantly more VA than did the TBI animals. There was a main effect for Stress, *F*(1,40) = 13.18, *p* < 0.001, η^2^ = 0.25, such that non-stressed animals had significantly more VA than did the stressed animals. There also was a significant Time × Group Interaction, *F*(3,40) = 6.73, *p* < 0.001, η^2^ = 0.33, a Time × Injury Interaction, *F*(1,40) = 9.08, *p* < 0.01, η^2^ = 0.19, and a Time × Injury × Stress Interaction, *F*(1,40) = 10.17, *p* < 0.01, η^2^ = 0.20. At day 3 post injury, there was a main effect for Group, *F*(3,40) = 7.61, *p* < 0.001, η^2^ = 0.36, such that Naïve animals had significantly more VA than did mTBI animals, Stress animals, and the S-mTBI animals. There was a main effect for Injury, *F*(1,40) = 11.81, *p* = 0.001, η^2^ = 0.23, such that non-injured animals had significantly more VA than did the TBI animals. There also was a main effect for Stress, *F*(1,40) = 13.34, *p* < 0.001, η^2^ = 0.25, such that non-stressed animals had significantly more VA than did stressed animals. At 5 days post injury, there was a main effect for Group, *F*(3,40) = 5.07, *p* < 0.01, η^2^ = 0.27, such that Naïve animals had significantly more VA than did S-mTBI animals. There also was a main effect for Stress, *F*(1,40) = 10.54, *p* < 0.01, η^2^ = 0.21, such that non-stressed animals had significantly more VA than did stressed animals.

#### Revised neurobehavioral severity scale

The NSS-R is a sensitive and reliable measure of sensory-motor responses in rodents (27, 28, 39). This measure models a clinical neurological exam of human patients and was based on several previous reports (29–32).

**Figure 2:**
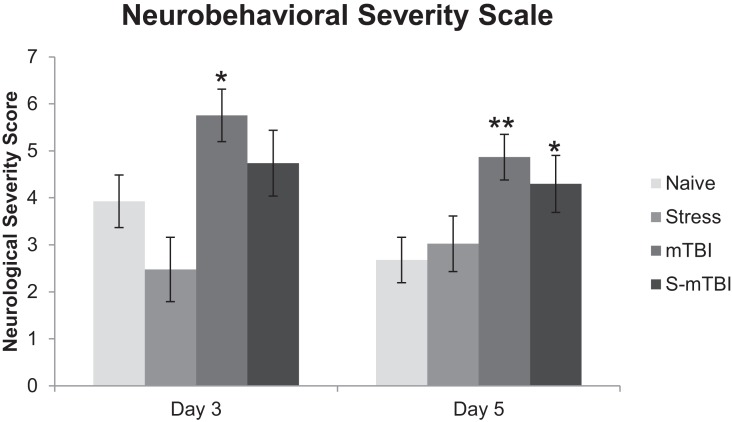
**Effects of stress, mTBI, or the combination on neurobehavioral function**. Neurobehavioral severity was determined by using a 10-item test with a score of 0, 1, 2, on each given task. Higher scores indicate more neurobehavioral impairment. Animals were tested at baseline (BL), and 3 and 5 days post injury. Neurobehavioral assessment post injury was covaried for baseline measurements due to differences at baseline between groups. *p < 0.05, **p < 0.01 vs. Naïves, respectively.

Figure 2 presents the neurobehavioral severity data (NSS-R; where higher scores indicate more sensorimotor functional impairment) of the animals. Overall, there was a main effect for Group, *F*(3,49) = 5.99, *p* < 0.001, η^2^ = 0.27, such that Naïve animals had significantly lower NSS-R scores than did mTBI animals. There also was a main effect for Injury, *F*(1,49) = 14.97, *p* < 0.001, η^2^ = 0.23, such that non-injured animals had significantly lower NSS-R scores than did TBI animals. Similarly, at 3 days post injury, there was a main effect for Group, *F*(3,49) = 4.23, *p* < 0.01, η^2^ = 0.21, such that Naïve animals had significantly lower NSS-R scores than did mTBI animals. There also was a main effect for Injury, *F*(1,49) = 10.53, *p* < 0.01, η^2^ = 0.18, such that non-injured animals had significantly lower NSS-R scores than did TBI animals. At 5 days post injury, there was a main effect for Group, *F*(3,49) = 4.72, *p* < 0.01, η^2^ = 0.22, such that Naïve animals had significantly lower NSS-R scores than did mTBI animals and S-mTBI animals. There also was a main effect for Injury, *F*(1,49) = 10.09, *p* < 0.01, η^2^ = 0.17, such that non-injured animals had significantly lower NSS-R scores than did TBI animals.

#### Acoustic startle response with and without pre-pulse

The ASR with and without pre-pulse are whole body behavioral responses believed to index information processing (40) and possibly attention (41–43).

**Figure 3:**
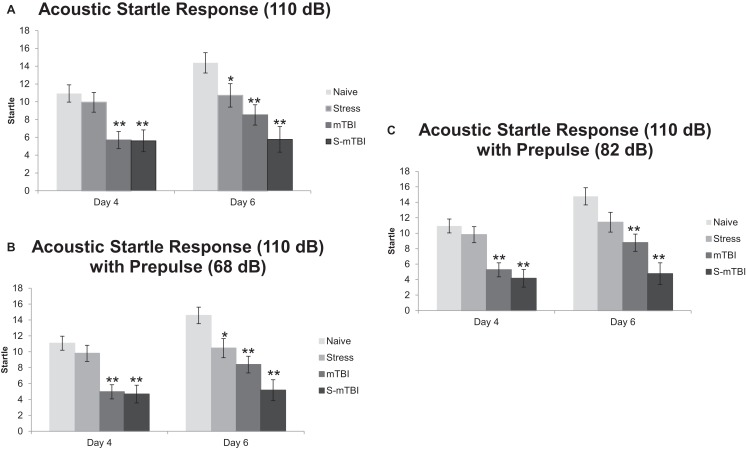
**Effects of stress, mTBI, or the combination on acoustic startle response (ASR) with and without pre-pulse (a measure of attention)**. **(A)** ASR at 110 dB alone throughout the experiment. **(B)** ASR at 110 dB with a 68-dB pre-pulse throughout the experiment. **(C)** ASR at 110 dB with an 82-dB pre-pulse throughout the experiment. Animals were measured at baseline (BL), and at 4 and 6 days post injury. *p < 0.05, **p < 0.01 vs. Naïves, respectively.

Figure 3A presents the ASR data with a tone of 110 dB. Overall, there was a main effect for Time, *F*(1,49) = 9.41, *p* < 0.01, η^2^ = 0.16, such that animals 4 days post injury had significantly lower startle to the tone than did animals at 6 days post injury. There also was a main effect for Group, *F*(3,49) = 9.21, *p* < 0.001, η^2^ = 0.36, such that Naïve animals had significantly higher startle to the tone than did mTBI animals and S-mTBI animals. There was a main effect for Injury, *F*(1,49) = 23.29, *p* < 0.001, η^2^ = 0.32, such that non-injured animals had higher startle responses than did TBI animals. There also was a significant Time × Stress Interaction, *F*(1,49) = 6.88, *p* < 0.050, η^2^ = 0.12. At 4 days post injury, there was a main effect for Group, *F*(3,49) = 7.25, *p* < 0.001, η^2^ = 0.31, such that Naïve animals had significantly more startle to the tone than did mTBI animals and S-mTBI animals. There also was a main effect for Injury, *F*(1,49) = 20.00, *p* < 0.001, η^2^ = 0.29, such that non-injured animals had significantly higher startle to the tone than did TBI animals. At 6 days post injury, there was a main effect for Group, *F*(3,49) = 8.42, *p* < 0.001, η^2^ = 0.34, such that Naïve animals had significantly higher startle to the tone than did Stress animals, mTBI animals, and S-mTBI animals. There was a main effect for Injury, *F*(1,49) = 18.25, *p* < 0.001, η^2^ = 0.27, such that non-injured animals had more startle to the tone than did TBI animals. There also was a main effect for Stress, *F*(1,49) = 6.40, *p* < 0.050, η^2^ = 0.12, such that non-stressed animals had more startle to the tone than stressed animals.

Figure 3B represents the ASR data with a tone of 110 dB and a pre-pulse of 68 dB (heard 100 ms before the tone). Similar results were found here as with the 110-dB tone alone. Overall, there was a main effect for Time, *F*(1,49) = 4.66, *p* < 0.05, η^2^ = 0.09, such that animals at 4 days post injury had significantly less startle than animals at 6 days post injury. There was a main effect for Group, *F*(3,49) = 15.15, *p* < 0.001, η^2^ = 0.48, such that Naïve animals had significantly more startle than did Stress animals, mTBI animals, and S-mTBI animals. There was a main effect for Injury, *F*(1,49) = 37.79, *p* < 0.001, η^2^ = 0.44, such that non-injured animals had significantly more startle than did TBI animals. There was a main effect for Stress, *F*(1,49) = 5.77, *p* < 0.050, η^2^ = 0.11, such that non-stressed animals had significantly more startle than did stressed animals. There also was a significant Time × Stress Interaction, *F*(1,49) = 6.99, *p* < 0.050, η^2^ = 0.13. At 4 days post injury, there was a main effect for Group, *F*(3,49) = 11.85, *p* < 0.001, η^2^ = 0.42, such that Naïve animals had significantly more startle than did mTBI animals and S-mTBI animals. There also was a main effect for Injury, *F*(1,49) = 32.73, *p* < 0.001, η^2^ = 0.40, such that non-injured animals had significantly more startle than did TBI animals. At 6 days post injury, there was a main effect for group, *F*(3,49) = 11.81, *p* < 0.001, η^2^ = 0.42, such that Naïve animals had significantly more startle than did stress animals, mTBI animals, and S-mTBI animals. There was a main effect for Injury, *F*(1,49) = 24.59, *p* < 0.001, η^2^ = 0.33, such that non-injured animals had significantly more startle than did TBI animals. There also was a main effect for Stress, *F*(1,49) = 10.02, *p* < 0.01, η^2^ = 0.17, such that non-stressed animals had significantly more startle than did stressed animals.

Figure 3C represents the ASR data with a tone of 110 dB and a pre-pulse of 82 dB (heard 100 ms before the tone). Similar results were found here as with the 110-dB tone alone. Overall, there was a main effect for Time, *F*(1,49) = 7.18, *p* < 0.01, η^2^ = 0.13, such that animals at 4 days post injury had significantly less startle than animals at 6 days post injury. There was a main effect for Group, *F*(3,49) = 15.03, *p* < 0.001, η^2^ = 0.48, such that Naïve animals had significantly more startle than did mTBI animals and S-mTBI animals. There was a main effect for Injury, *F*(1,49) = 39.10, *p* < 0.001, η^2^ = 0.44, such that non-injured animals had significantly more startle than did TBI animals. There was a main effect for Stress, *F*(1,49) = 6.27, *p* < 0.05, η^2^ = 0.11, such that non-stressed animals had significantly more startle than did stressed animals. There also was a significant Time × Stress Interaction, *F*(1,49) = 4.80, *p* < 0.05, η^2^ = 0.090. At 4 days post injury, there was a main effect for Group, *F*(3,49) = 11.31, *p* < 0.001, η^2^ = 0.41, such that Naïve animals had significantly more startle than did mTBI animals and S-mTBI animals. There also was a main effect for Injury, *F*(1,49) = 32.01, *p* < 0.001, η^2^ = 0.40, such that non-injured animals had significantly more startle than did TBI animals. At 6 days post injury, there was a main effect for Group, *F*(3,49) = 11.42, *p* < 0.001, η^2^ = 0.41, such that Naïve animals had significantly more startle than did mTBI animals and S-mTBI animals. There was a main effect for Injury, *F*(1,49) = 26.17, *p* < 0.001, η^2^ = 0.35, such that non-injured animals had significantly more startle than did TBI animals. There also was a main effect for Stress, *F*(1,49) = 8.90, *p* < 0.01, η^2^ = 0.15, such that non-stressed animals had significantly more startle than did stressed animals. Similar results were found with 120 dB with and without pre-pulses (data not shown).

### Western blot data

#### Prefrontal cortex

**Figure 4:**
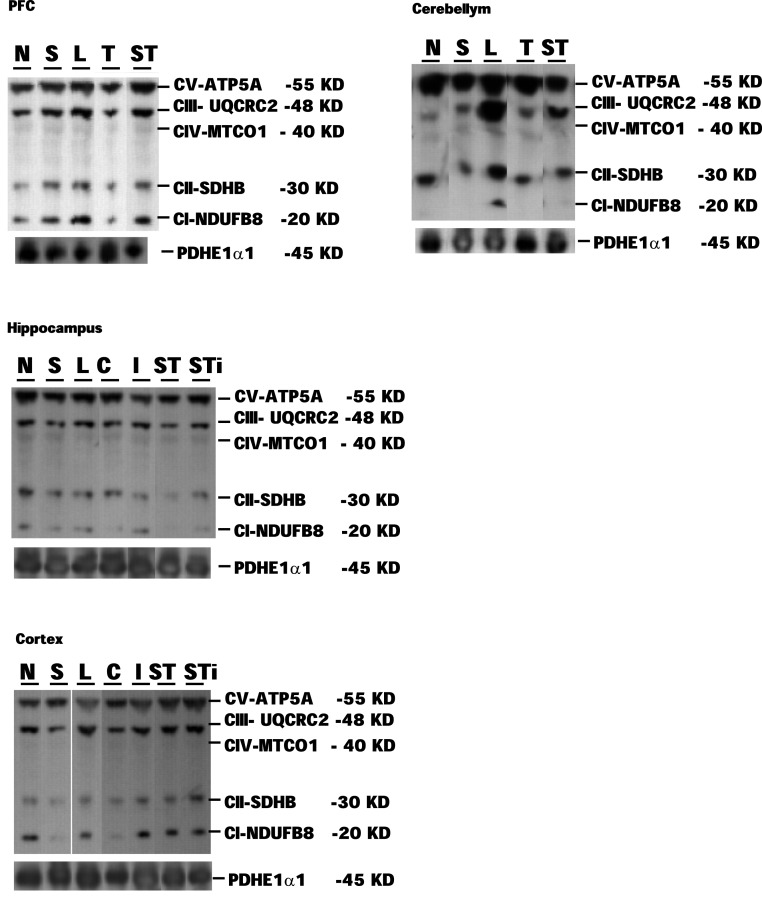
**Representative samples of Western blotting of CI, CII, CIII, CIV, CV, and PDHE1α1 protein bands in the tissue homogenates of rat prefrontal cortex (PFC), cerebellum, hippocampus, and cerebral cortex collected at 7 days post mTBI**. Contralateral and ipsilateral hippocampus and cerebral cortex were collected for mTBI and S-mTBI animals. Twenty micrograms of total proteins were resolved on SDS-PAGE gel and incubated with the primary antibodies against each protein. N, Naïves; S, Stress; T, mTBI; ST, stress followed by mTBI; C, contralateral mTBI; I, ipsilateral mTBI; ST, contralateral S-mTBI; STi, ipsilateral S-mTBI.

**Figure 5:**
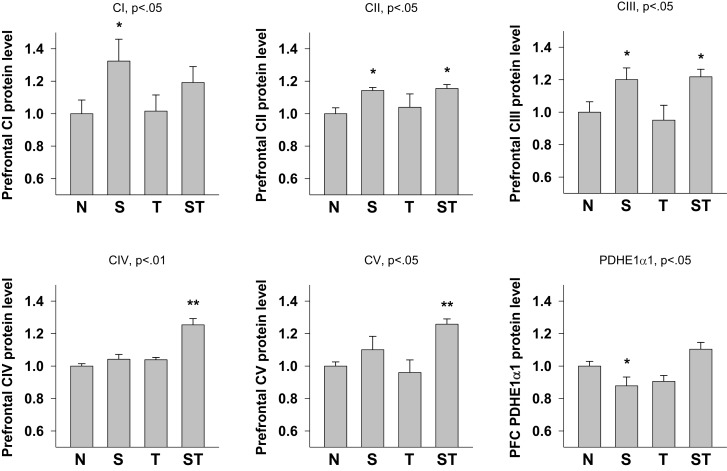
**Semi-quantitative determination of the Western blotting protein bands density of ETC subunits CI, CII, CIII, CIV, CV proteins, and PDHE1α1 protein expressed in rat prefrontal cortex (PFC) 7 days post mTBI**. N, Naïves; S, Stress; T, mTBI; ST, stress followed by mTBI. Results are presented as the fold change relative to the Naïves (=1). *p < 0.05, **p < 0.01 vs. Naïves, respectively.

Expression levels of mitochondrial proteins in Stress and mTBI groups was reduced, whereas S-mTBI increased PDHE1α1 protein level in the prefrontal cortex (Figure 4). One-way ANOVA revealed significant effects of repeated stress and mTBI treatment on ETC CI (*p* < 0.05), CII (*p* < 0.05), CIII (*p* < 0.05), CIV (*p* < 0.01), CV (*p* < 0.05), and PDHE1α1 (*p* < 0.05) protein levels (Figure 5). LSD *post hoc* showed that when compared with Naïves, Stress, and S-mTBI animals had significant enhancing effects on CI, CII, and CIII. S-mTBI animals also had enhancing effects on CIV and CV protein levels. In contrast, mTBI treatment alone did not affect ETC subunit expression in the prefrontal cortex.

#### Cerebellum

**Figure 6:**
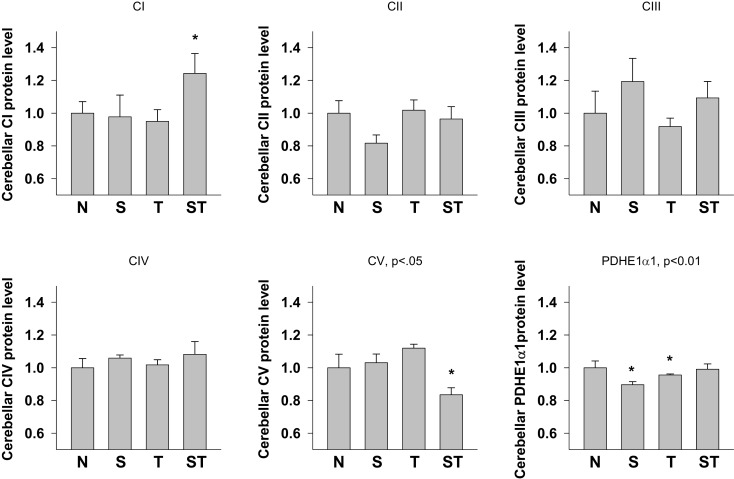
**Semi-quantitative determination of the Western blotting protein bands density of ETC subunits CI, CII, CIII, CIV, CV proteins, and PDHE1α1 protein expressed in rat cerebellum 7 days post mTBI**. N, Naïves; S, 3 days repeated stress; T, mTBI; ST, stress followed by mTBI. Results are presented as the fold change relative to the Naïves (=1). *p < 0.05, **p < 0.01 vs. Naïves, respectively.

One-way ANOVA showed significant effects of Stress and S-mTBI on cerebellar CI (*p* < 0.05), CV (*p* < 0.05) and PDHE1α1 (*p* < 0.05) expression in rat cerebellum (Figures 4 and 6). LSD *post hoc* showed that when compared with Naïves, cerebellar CI protein level increased significantly (*p* < 0.05) whereas CV protein decreased at a trend level (*p* < 0.01) in S-mTBI-treated animals. Cerebellar PDHE1α1 protein level decreased in the Stress and mTBI groups compared to the Naïve group.

#### Hippocampus

**Figure 7:**
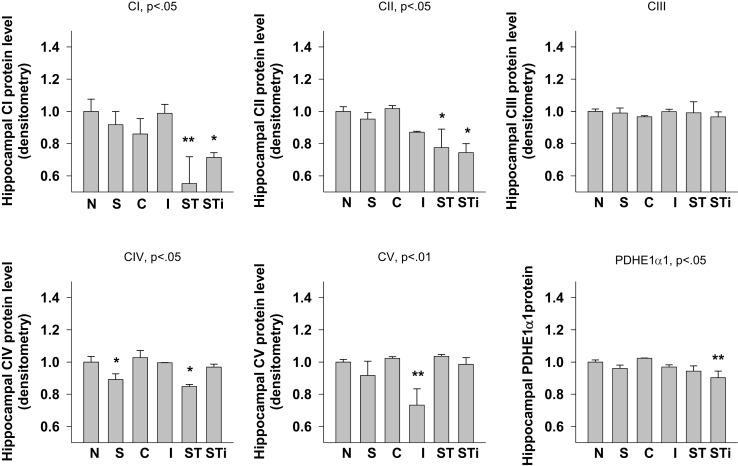
**Semi-quantitative determination of the Western blotting protein bands density of ETC subunits CI, CII, CIII, CIV, CV proteins, and PDHE1α1 protein expressed in rat hippocampus 7 days post mTBI**. N, Naïves; S, 3 days repeated stress; T, mTBI; ST, stress followed by mTBI; C, contralateral mTBI; I, ipsilateral mTBI; ST, contralateral S-mTBI; STi, ipsilateral S-mTBI. Results are presented as the fold change relative to the Naïves (=1). *p < 0.05, **p < 0.01 vs. Naïves, respectively.

One-way ANOVA showed significant effects of Stress, mTBI, and S-mTBI on CI (*p* < 0.05), CII (*p* < 0.05), CIV (*p* < 0.05), CV (*p* < 0.01), and PDHE1α1 (*p* < 0.05) protein levels in the hippocampus. LSD *post hoc* showed that, when compared with the Naïve hippocampus, CI and CII proteins in the contralateral and ipsilateral hippocampus of the S-mTBI animals decreased significantly. Complex IV protein levels in the hippocampus of Stress animals and contralateral hippocampus of S-mTBI animals, as well as CV protein level in the ipsilateral hippocampus of mTBI animals also decreased significantly. PDHE1α1 protein level in the ipsilateral hippocampus of S-mTBI animals also decreased significantly (*p* < 0.01) (Figures 4 and 7).

#### Cerebral cortex

**Figure 8:**
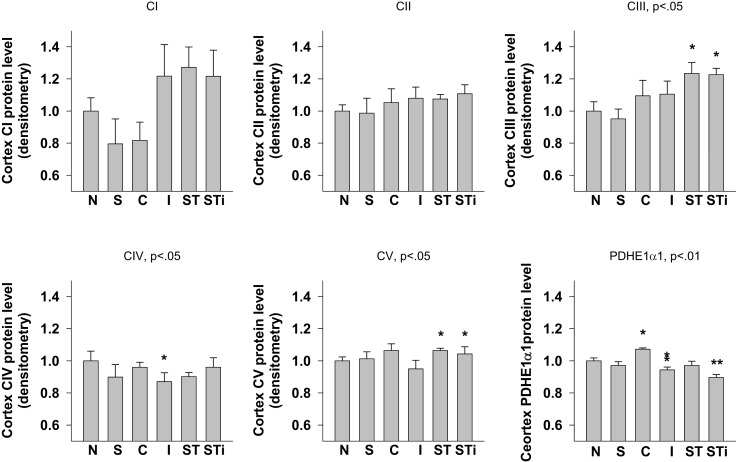
**Semi-quantitative determination of the Western blotting protein bands density of ETC subunits CI, CII, CIII, CIV, CV proteins, and PDHE1α1 protein expressed in rat cerebral cortex 7 days post mTBI**. N, Naïves; S, 3 days repeated stress; T, mTBI; ST, stress followed by mTBI; C, contralateral mTBI; I, ipsilateral mTBI; ST, contralateral S-mTBI; STi, ipsilateral S-mTBI. Results are presented as the fold change relative to the Naïves (=1). *p < 0.05, **p < 0.01 vs. Naïves, respectively.

One-way ANOVA showed significant effects of TBI treatment on CIII (*p* < 0.05), CIV (*p* < 0.05), CV (*p* < 0.01), and PDHE1α1 (*p* < 0.01) protein expression in the cerebral cortex (Figures 4 and 8). LSD *post hoc* revealed that when compared with the Naïve group, CIII protein level was significantly higher in the contralateral and ipsilateral cortex of S-mTBI animals (*p* < 0.05), CIV protein level was significantly lower in the ipsilateral cortex of mTBI animals (*p* < 0.05), and CV protein level was significantly higher in the cortex of S-mTBI animals (*p* < 0.05). PDHE1α1 was significantly higher in the contralateral cortex of mTBI animals (*p* < 0.05) but lower in the ipsilateral cortex of mTBI (*p* < 0.05) and S-mTBI animals (*p* < 0.01).

## Discussion

The prevalence of post-concussive syndrome associated with increased anxiety and memory deficit are particularly high among military casualties of the Iraq and Afghanistan wars (5). The role of psychological stress in the battlefield is very important on the outcome of TBI. Currently, the overlapping depressive symptomology of PTSD and mTBI present a major diagnostic challenge and dilemma for clinicians. In this study, we have dissected the neurobehavioral symptoms and altered brain metabolic pathways following stress or mTBI alone, and combined effect of stress and mTBI in rats. The key findings of this study are (1) animal exposure to the repeated stress or mTBI alone resulted in an early and short term increase in anxiety and impaired memory, (2) these symptoms persisted for a long time in animals with combined stress and mTBI, and (3) abnormal mitochondrial ETC and PDH enzyme expressions in different parts of the brain were seen in all animals with stress with or without brain injury confirming the altered cellular metabolic pathways due to stress or mTBI (44, 45).

### Behavioral effects of stress, mTBI, or stress with mTBI

The presence of repeat stress in our rat model had little effect on sensorimotor responses, but significant decrease in startle responses, with and without pre-pulse at day 6. These findings suggest that stress initially decrease movement and temporary increase in depression-related behaviors. These findings are similar to the previous published report in which rat exposure to fear only caused temporary increased in anxiety and impaired memory (46). In contrast, our startle data reveals that information processing was not immediately affected by stress, but became abnormal over time suggesting the progression of secondary brain injury in our rat model of repeat stress. Similarly, patients with PTSD also frequently display increased arousal, which is manifested by irritability, attention deficit, and disturbed sleep (3, 47). These observations confirm the similarities of depression like symptoms in our rat repeat stress model and patients with PTSD.

Brain injury alone caused a significant decline in sensorimotor function and startle responses throughout the experiment when compared with naïves animals. These observations indicate that even mTBI initially triggers the depression like behaviors that recovered within 6 days following injury, but the poor sensorimotor function persisted. Similar cognitive dysfunctions have also been published in rat models with controlled cortical impact injury, lateral and midline FP injury, and blast (48). In humans also, cognitive dysfunctions and impaired memory are the common clinical manifestations of mTBI that have not been widely studied in animal models (49). Therefore, functional neurobehavioral responses to mTBI in this study are important for future diagnosis and treatment of mTBI.

The combination of stress and brain injury appeared to produce an additive effect on activity, sensorimotor function, and startle responses. A significant decreased the horizontal and VA, sensorimotor functions and startle responses were noted throughout the experiment in animals with combined stress and mTBI. The additional behavioral findings in our study confirms that the combination of injury and repeated stress were particularly disruptive. Naïve animals showed the appropriate habituation expected over the course of the experiment with regards to OFA and NSS-R (i.e., the animals’ activity or score decreased over time). It is also worth noting that animals were also tested using the rotarod (data not shown), to test for motor deficits, and while TBI decreased the time the animals were able to balance, stress improved the animals’ balance. This finding helps in the interpretation of the OFA results, indicating that the combination of stress and mTBI did not cause any motor deficits, therefore the decrease in horizontal and VA can be interpreted as deteriorated general health and depressive-related behaviors.

### Mitochondrial effects of stress, mTBI, or stress with mTBI

The specific role of primary mechanisms in stress, mTBI, or combined effects of stress with mTBI is difficult to assess in clinical cases. However, postmortem analysis of brain tissue from patients with PTSD and/or TBI indicated the involvement of mitochondria in neuronal cell death and hippocampus atrophy (44, 45). We believe this is the first study to examine the proteins responsible for mitochondrial energy producing pathways in response to stress, mTBI, or stress with mTBI.

As with the behavioral effects, the stress and injury manipulations have significant measureable effects on PDH and ETC expression in different parts of the brain. These findings extend our previous findings of altered mitochondrial PDH expression and activity after TBI (12, 13). Similar to our findings of neurobehavioral effects of combined stress and mTBI, present experiment indicate that the combination of repeated stress and mTBI had the most effects on mitochondrial PDH and ETC subunit expression compared with stress or mTBI alone. Therefore, the parallel effects of combined stress and injury on behavioral and brain ETC activity are noteworthy and merits further investigation.

The PFC is known to exert a powerful inhibitory effect on amygdala activity and plays an important role in fear extinction (50, 51). The increased ETC subunit expression in the PFC of stressed animals (especially in the stress plus injury animals) could be associated with increased inhibition of amygdala activation, altered fear memory and affect the reorganization of interconnection and inter-regulation between the PFC and limbic circuits to alter endurance and resistance from further stress (52). The region-specific increase of ETC subunits expression in the PFC of Stress and S-mTBI animals is also in agreement with the recent report that chronic stress sensitizes the frontal cortex to the release of cytochrome *c* (CIV) from the mitochondria of male rats. While the relevance of increased PFC in ETC expression in an animal model of repeated stress with brain injury (that may model PTSD with mTBI) remains to be validated. Recent brain imaging studies indicate that combat-exposed war veterans with PTSD and mTBI with high risk for suicide also had hyperactivation of the PFC and anterior cingulate during error processing compared to non-suicidal PTSD with mTBI veterans (53).

Although the mechanism and biological significance of pre-existing stress on the severity of brain injury remains obscure, the enhanced ETC expression may also reflect a compensatory mechanism for increased energy demand of the injured brain due to increased neuronal activity in several brain regions. This data is also in line with the reported up-regulation of cannabinoid receptor (CBR) expression, an important mediator of energy metabolism in the PFC of juvenile male rats after repeated stress (54). In contrast to the increase ETC complexes in the PFC, the expression of CI, CII, CIV, and PDHE1α1 were significantly reduced in the hippocampus of the stress plus injury animals. CV also was decreased in the cerebellum of the stress plus injury animals. These results suggest an increased vulnerability of a repeatedly stressed hippocampus to the detrimental effects of mTBI in terms of ETC complex expression and activity. Reduced ETC and PDHE1α1 expression are consistent with reports that inhibition or deficits of mitochondrial ETC complexes are associated with increased ROS production, increased oxidative damage, and apoptotic cell death in the hippocampus after TBI (55–61). These findings corroborate the observations of Opii et al. (62) indicating that, following TBI, several mitochondrial proteins involved in energy producing pathways are modified or oxidatively damaged in different parts of the brain, which may eventually cause cell death and brain atrophy (45). Therefore, the identification of these proteins in response to stress alone or stress followed by mTBI may provide new insights into the brain cell metabolic mechanisms and possible therapeutic interventions after mTBI.

The hippocampus is highly vulnerable to brain injury in both animal models of TBI and humans with TBI, and the hippocampus volume is also reduced in patients with PTSD (63–65). The hippocampus undergoes atrophy and contributes to the chronic memory deficits in the weeks to months following a mTBI (66, 67). Other studies reported that alterations in hippocampus ETC level is associated with aging and increased oxidative damage in mice brains (68) and with Alzheimer’s disease (69), a neurodegenerative disorder common among TBI patients (70, 71).

#### Summary

The behavioral and brain protein data support a greater impact of combined stress plus brain injury than mTBI or stress alone on neurobehavioral function and brain mitochondrial ETC expression. Repeated stress exposure prior to TBI potentiated mitochondrial ETC subunit expression in the various brain regions and also potentiated several behavioral effects in rats. These results may explain the relationship between altered regional brain mitochondrial activity and functional outcomes in people with PTSD and mTBI. Repeated stress could have contributed to the high incidence of long-term neurologic and neuropsychiatric morbidity in military personnel with mTBI.

## Conflict of Interest Statement

The authors declare that the research was conducted in the absence of any commercial or financial relationships that could be construed as a potential conflict of interest.
